# Kinematic and Dynamic Vehicle Model-Assisted Global Positioning Method for Autonomous Vehicles with Low-Cost GPS/Camera/In-Vehicle Sensors

**DOI:** 10.3390/s19245430

**Published:** 2019-12-09

**Authors:** Haigen Min, Xia Wu, Chaoyi Cheng, Xiangmo Zhao

**Affiliations:** 1 The Joint Laboratory for Internet of Vehicles, Ministry of Education-China Mobile Communications Corporation, Xi’an 710064, China; 2 Information & Engineering School, Chang’an University, Xi’an 710064, China; viichelle_wu@chd.edu.cn (X.W.); chao_yi_cheng@163.com (C.C.)

**Keywords:** global positioning system, simultaneous localization and mapping, multi-sensor fusion, autonomous vehicle

## Abstract

Real-time, precise and low-cost vehicular positioning systems associated with global continuous coordinates are needed for path planning and motion control in autonomous vehicles. However, existing positioning systems do not perform well in urban canyons, tunnels and indoor parking lots. To address this issue, this paper proposes a multi-sensor positioning system that combines a global positioning system (GPS), a camera and in-vehicle sensors assisted by kinematic and dynamic vehicle models. First, the system eliminates image blurring and removes false feature correspondences to ensure the local accuracy and stability of the visual simultaneous localisation and mapping (SLAM) algorithm. Next, the global GPS coordinates are transferred to a local coordinate system that is consistent with the visual SLAM process, and the GPS and visual SLAM tracks are calibrated with the improved weighted iterative closest point and least absolute deviation methods. Finally, an inverse coordinate system conversion is conducted to obtain the position in the global coordinate system. To improve the positioning accuracy, information from the in-vehicle sensors is fused with the interacting multiple-model extended Kalman filter based on kinematic and dynamic vehicle models. The developed algorithm was verified via intensive simulations and evaluated through experiments using KITTI benchmarks (A project of Karlsruhe Institute of Technology and Toyota Technological Institute at Chicago) and data captured using our autonomous vehicle platform. The results show that the proposed positioning system improves the accuracy and reliability of positioning in environments in which the Global Navigation Satellite System is not available. The developed system is suitable for the positioning and navigation of autonomous vehicles.

## 1. Introduction

Continuous, accurate and high-integrity positioning information is critical for the operation of autonomous vehicles, including path planning and motion control functions. Several concerns and challenges remain regarding the positioning and navigation systems of autonomous vehicles, including the following:

(1) Perception in extreme weather: It is difficult for visual or light detection and ranging (LiDAR) sensors to identify lane lines when the road is covered with water or snow. Furthermore, in open areas, a sufficient number of road markers cannot be detected. A high-precision Global Navigation Satellite System (GNSS) is not affected by these factors and can thus provide improved perception to some extent.

(2) Safety and comfort: Due to the performance limits of vehicular sensors like cameras and LiDAR, the vehicle sometimes cannot detect the road curvature or slope. This can result in safety and comfort concerns related to sudden braking or sharp turns.

(3) Vehicle-to-everything applications: When crossing an intersection, the vehicle can accurately determine its own position and share it with other vehicles through vehicle-to-vehicle communication or vehicle-to-infrastructure communication, which are fundamental technologies for fully automated vehicle scheduling.

(4) Computational and storage load: Global positioning information contributes to the initialisation process of high-definition (HD) maps. There is no need for a vehicle to load the entire HD map at once; the vehicle only needs to load a certain part of map from the map database based on the vehicle’s current position, greatly reducing the storage and computational load.

GNSS includes four main systems, namely the American Global Positioning System (GPS), the Russian Glonass, the European Galileo and Chinese BeiDou [1,2,3]. Taking the GPS for example, it can provide positioning information in the global coordinate system, while a civilian GPS has a low measurement frequency and a large error; thus, it cannot provide the accuracy and stability needed for autonomous vehicles. The error in the GPS is determined by three factors: (1) satellite orbit error and clock error at the launch stage; (2) refraction error during signal propagation, which is unavoidable because of the non-uniform ionosphere and troposphere; and (3) clock error and noise during signal transmission. In complex urban environments, the error in the GPS can reach tens of metres. To improve the accuracy of a common GPS, differential and real-time kinematic techniques have been developed. Although these strategies can improve the GPS positioning accuracy to the decimetre or centimetre level, the GPS signals are still blocked by tall buildings, tunnels, mountain roads, dense foliage and so on.

Some techniques can provide relatively precise positioning information even though they estimate the position in local coordinates. For example, simultaneous localisation and mapping (SLAM) involves constructing a model of the environment (the map) and estimating the state of the moving robot [4]. SLAM methods include visual SLAM (V-SLAM) and LiDAR SLAM. The map constructed by SLAM contains abundant driving assistance information, including road data and data on stable objects. Using these data, the navigation system can accurately identify the terrain, road contours and other information to guide the vehicle. The drawback of SLAM is that the map is difficult to save and update, and natural accumulative error cannot be eliminated. Thus, SLAM is a good choice for a short-distance positioning system rather than for a long-distance one. The in-vehicle sensor data (e.g., vehicle speed and steering angle) are easily obtained via a controller area network (CAN). Positioning based on in-vehicle sensors is a type of dead reckoning (DR) localisation, which results in a large amount of error in high-speed and tyre-slip scenarios [5]. Inertial measurement units (IMUs) can provide continuous pose information and are not affected by the environment. However, IMUs are also prone to integral errors due to drift error over time [6]. Moreover, precision-calibrated fibre-optic gyroscopes are too expensive for use in commercial autonomous vehicles. Positioning methods based on wireless networks calculate positions using information such as the time difference of the signal propagation or the signal strength. However, these methods are subject to errors caused by communication delays and the multipath effect [7]. An appropriate vehicle model that can cover various driving conditions will improve the accuracy of the positioning system [8].

Taking advantage of the complementary characteristics of a GPS, V-SLAM and in-vehicle sensors, this paper provides a continuous, accurate and high-integrity positioning method suitable for use in various challenging environments (e.g., urban areas with tall buildings, tunnels and overpasses). The new method was developed in consideration of computational complexity, cost and unexpected noise. The tightly coupled architecture was designed to update the global position measurement even when less than four satellites are available. In open spaces, image features are too sparse to stably and accurately estimate the position with V-SLAM; a GPS is more appropriate in this situation. In indoor environments where GPS signals are easily blocked, cameras can detect objects and provide abundant information. In this paper, two novel algorithms, image singular value decomposition (ISVD) and statistic filter of feature space displacement (SFFSD), were proposed to ensure the robustness and accuracy of the V-SLAM procedure in the local environment. Next, V-SLAM and a GPS were calibrated with regression analysis and error correction, and the calibration result was used to update the vehicle model-based positioning information. To account for various driving conditions, a kinematic vehicle model and a dynamic vehicle model were considered to refine the calibration result. Finally, we assessed the performance of the proposed algorithm from a practical perspective using simulations, KITTI benchmarks and experiments.

This paper is organised as follows. Section 2 provides a brief overview of related work. Section 3 discusses the problem framework and proposed positioning strategies, including the vehicle model set, V-SLAM and its calibration with a GPS and the interacting multiple model filter. Section 4 details the experiments and evaluations, and Section 5 presents the conclusions.

## 2. Related Work

Several positioning methods have been developed over the last five decades. Some of these methods are summarised below.

### 2.1. Global Navigation Satellite System (GNSS) Localisation

GNSS refers to all satellite navigation systems, including global and regional systems (e.g., the American GPS, the Russian Glonass system, the European Galileo system and the Chinese Beidou satellite navigation system) and related enhancement systems (e.g., the American Wide-Area Augmentation System, the European Geostationary Navigation Overlay System and the Japanese Multi-functional Transport Satellite Augmentation System) [9]. The GPS is currently the most common GNSS system due to its low cost, wide applicability and weather resistance. However, the GPS has several limitations. First, the satellite signals may be blocked in tunnels, mountain roads and streets surrounded by tall buildings, preventing the GPS receiver from receiving satellite signals. Second, satellite-based localisation performance is affected by several factors, including satellite clock drift, transmission through the atmosphere and multipath propagation. The typical localisation accuracy of the GPS is between 10 and 20 m, significantly larger than the width of a typical traffic lane. To reduce the error caused by clock drift and atmospheric transmission, a ground-based differential GPS (DGPS) was proposed. DGPS can significantly reduce the position estimation error, improving the localisation accuracy to several metres. However, the error caused by multipath propagation cannot be mitigated, and the accuracy and reliability of localisation remain insufficient for applications, such as collision warnings, platooning and automatic parking.

### 2.2. DR Localisation

DR is a classic localisation technique that is independent of the GNSS. For an object moving in two-dimensional space, if its initial position and all displacements at previous time points are known, the current position of the object can be calculated from the initial position and the cumulative displacement obtained by inertial sensors (e.g., odometers, gyroscopes, accelerometers and electronic compasses). There are two requirements for the implementation of DR localisation: (1) the initial position of the moving target must be known and (2) the distance and direction of the moving target at all moments must be obtained [10]. DR systems have several advantages, including high autonomy, high security, good resistance to radio interference and weather resistance. Furthermore, DR systems use only stand-alone inertial measurement components to calculate the position, speed and other navigation parameters. However, since DR localisation is an accumulative process, each estimated position of the target depends on the localisation of previous movements. Thus, the measurement and calculation errors accumulate over time, leading to continuous deterioration in accuracy. As a result, DR localisation is unsuitable for long-term operation. In addition, initial alignment in a DR system requires time, especially for position measurements [11].

### 2.3. Map Matching Localisation

Map matching is a software-based localisation correction method. By associating GPS localisation with road information in a digital map database, the vehicle position relative to the map can be determined [12]. Map matching applications are based on two assumptions: (1) all vehicles travel only on roads and (2) the accuracy of the digital map is higher than that of the estimated position of the vehicle on the road. When these conditions are met, the localisation trajectory is compared with the road information via an appropriate matching process to determine the road sections that the vehicle is most likely to travel along with the vehicle’s most likely position within these road sections. The performance of a map-matching algorithm depends strongly on the resolution of the digital map [13]. The digital map must have the correct network topology and high accuracy; otherwise, false matches will occur [14].

### 2.4. Mobile Radio-Based Localisation

Mobile radio-based localisation is a process of locating an object using a land-based wireless infrastructure. Generally, this process involves measuring the transmission parameters of radio waves (e.g., the difference in wave arrival time or phase or the variation in amplitude or frequency) travelling from known stationary objects to a moving target. Based on these parameters, the difference in distance between the known stationary objects and the target object, along with the moving direction of the target object, can be determined [15]. A common application of mobile radio localisation is the localisation of the mobile phone user by the American 911 telephone system. Other mobile radio-based localisation methods including localisation based on ultra-wideband and WiFi signals and cooperative localisation based on a Vehicular Ad-Hoc Network (VANET) [16,17,18]. However, mobile radio-based localisation requires an infrastructure with many roadside base stations, resulting in a high cost. Some researchers have incorporated SLAM into mobile radio-based localisation applications [19].

### 2.5. Vision/LiDAR-Based Localisation

Vision/LiDAR-based positioning has attracted considerable attention in recent years. Vision-based positioning has been widely explored because of its low cost and immunity to electromagnetic interference [20,21,22]. However, the resulting images are affected by changing light and image blurring (images captured during vehicle vibration or sharp turns), which break the projection relationship between the scene points and the image pixels. Most existing visual positioning methods suffer from a large cumulative error and non-real-time performance. Many studies [23,24,25] have reported the advantages of LiDAR-based positioning, including robustness in bad weather (variable light, rain, fog and snow). However, its cost and time-consuming nature restrict the large-scale application of LiDAR-based positioning. Global or local optimisation and loop-closure detection are tricky problems that need to be solved.

### 2.6. Multiple Sensor-Based Localisation

Approaches combining multiple sensor types are becoming the norm in practical positioning and navigation systems. Common multi-sensor systems combine a GPS and inertial navigation systems in loose-coupled or tightly coupled architectures [26]. However, professional and high-grade devices come with a high overall cost. In Jo et al. [8], a rule-based filter update method with a validation gate was adopted. The system did not update the GPS measurement when the number of satellites in use was four or less. That study completely ignored the fact that a positioning assessment based only on the number of satellites is not reliable, and the positioning result was affected by the GPS signal reflection and the multipath effect. Al Hage et al. [27] designed a tightly coupled GPS/odometer with faults diagnosis to mitigate GPS errors. Model-aided positioning was first applied in spaceflight position estimation, and its performance was evaluated. Some studies [5,8] have combined different in-vehicle sensors to improve the accuracy of the positioning system; however, most of these were designed for manned vehicles rather than driverless vehicles. In manned vehicles, metre- or sub-metre-level error, which results in occasional discontinuity or drift, is acceptable. In contrast, these situations are not acceptable in autonomous vehicles as they will seriously affect reliability and safety.

## 3. Proposed Fusion Positioning Strategy

**Figure 1:**
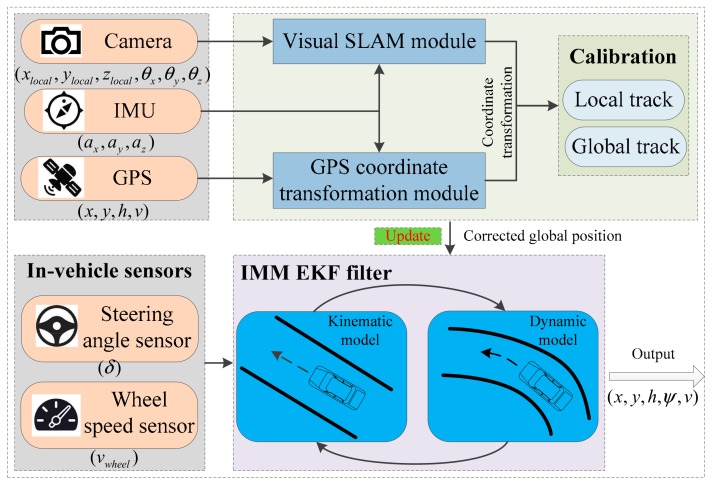
Structure of the overall algorithm architecture. GPS: global positioning system, IMU: inertial measurement unit, SLAM: simultaneous localisation and mapping.

In this section, we detail the proposed positioning strategy. Figure 1 shows the system structure. The vehicle models, which include kinematic and dynamic vehicle models, are explained first. Next, the V-SLAM algorithm and its calibration using a GPS track algorithm are presented. The, the calibration result used to update measurements are given. Finally, the in-vehicle sensors and vehicle models and how they were employed to refine the calibration result are discussed.

### 3.1. Vehicle Modelling

The incorporation of kinematic and dynamic vehicle models in the positioning method can compensate for a GPS signal loss. The kinematic vehicle model is suitable for low-speed and low-slip driving conditions, while the dynamic vehicle model is suitable for high-speed and large-slip motion. Herein, a vehicle motion model that combines the kinematic vehicle model and the dynamic vehicle model is established to account for numerous driving conditions.

#### 3.1.1. Kinematic Vehicle Model

**Figure 2:**
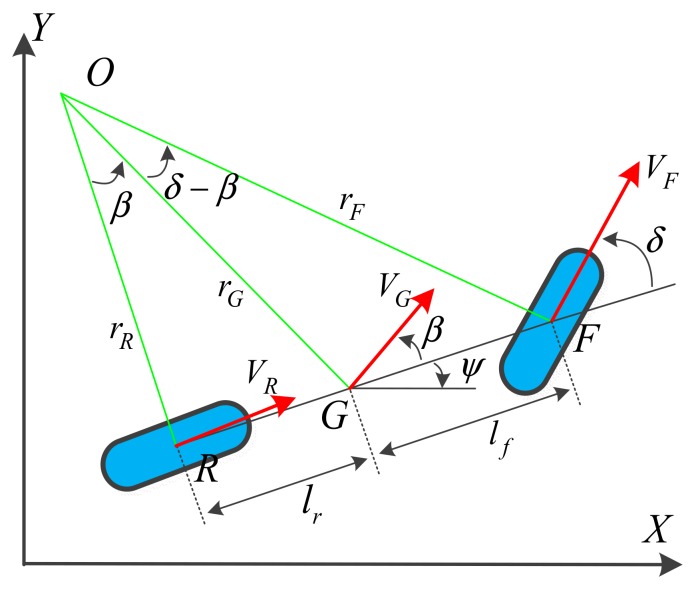
Kinematic vehicle model.

Although a rigid dynamic vehicle model is optimal for practical situations, the many degrees of freedom make the model complex, and the multi-parameter coupling is unfavourable for use in integrated navigation systems. According to the structure and characteristics of a front-wheel-steering vehicle, a four-wheeled vehicle can be simplified into a two-wheeled bicycle model [28]. As shown in Figure 2, in the bicycle model, the front and rear wheels are expressed by single wheels. In Figure 2, *G* is the vehicle’s centre of gravity; *O* is the centre of vehicle current motion; *V_G_* is the velocity of *G*; δ is the front wheel deflection; β is the corresponding slip angle of *G;* ψ and ψ + β are the heading and course angle of the vehicle, respectively; and *l_f_* and *l_r_* are the distance from *G* to the front (point *F*) and rear (point *G*) wheel, respectively; $r_{F}$, $r_{G}$, and $r_{R}$ are the radius from the motion centre *O* to the front wheel centre *F*, the gravity centre *G* and the rear centre *R*, respectively.

The kinematic vehicle model assumes that no slip occurs between the ground and the wheels, which is accurate for vehicles moving at low speeds. In this case, the velocity directions at points *F* and *R* (*V_F_* and *V_R_*, respectively) are consistent with the directions of the front and rear wheels, respectively. The kinematic relationship described above can be described using Equations (1)–(5):

(1) $$\dot{X}=V_{G}\cos(\psi +\beta ),$$

(2) $$\dot{Y}=V_{G}\sin(\psi +\beta ),$$

(3) $$\dot{\psi }=V_{G}\cos(\beta )\tan(\delta )/\left(l_{f}+l_{r}\right),\;$$

(4) $${\dot{V}}_{G}=a,$$

(5) $$\beta =\tan^{-1}\left(\frac{l_{r}}{l_{f}+l_{r}}\tan(\delta )\right).$$

#### 3.1.2. Dynamic Vehicle Model

**Figure 3:**
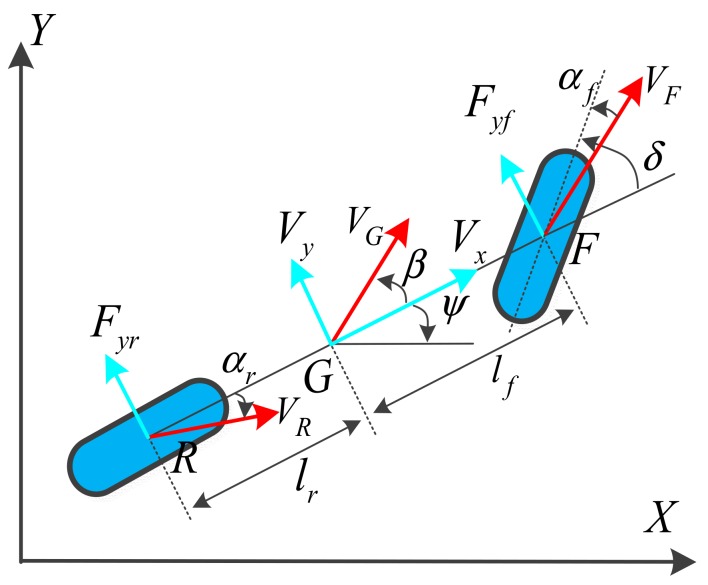
Dynamic vehicle model.

Considering vehicle slip and lateral velocity caused by changes in road conditions, vehicle load, wind speed/direction, etc., a dynamic vehicle is introduced to account for cases of high-speed motion in which the vehicle slides laterally but does not completely roll [29,30]. As shown in Figure 3, the tyre-slip angle is the angle between the wheel speed vector and the longitudinal wheel axis. Here, we assume that the tyre-slip angle is proportional to the lateral force acting on the tyre.

Equations (6) and (7) were derived according to the force and motion state of *G*:

(6) $$\sum F_{y}=F_{yf}+F_{yr}=ma_{y}=m({\dot{V}}_{y}+V_{x}r),$$

(7) $$\sum M_{z}=l_{f}-l_{r}F_{yr}=I_{z}\ddot{\psi },$$

where *m* is the mass of the vehicle; r is the velocity of heading angular ψ; *I_z_* is the inertial yaw moment; and *V_x_* and *V_y_* are the longitudinal and lateral velocities of the vehicle, respectively.

As shown in Figure 3, the dynamic vehicle model assumes that the tyre-slip angle is proportional to the lateral force that acts on the tyre:

(8) $$F_{yf}=2C_{f}\alpha_{f}\approx 2C_{f}\left(\beta +\frac{l_{f}\dot{\psi }}{V_{x}}-\delta \right),$$

(9) $$F_{yr}=2C_{r}\alpha_{r}\approx 2C_{r}\left(\beta -\frac{l_{r}\dot{\psi }}{V_{x}}\right),$$

where α*_f_* and α*_r_* are the slip angles relative to the front and rear wheels, respectively; and *C_f_* and *C_r_* are the cornering stiffnesses of the front and rear tyres, respectively.

By combining Equations (6) and (7) with Equations (8) and (9), the vehicle motion can be estimated by the dynamic vehicle model as follows:

(10) $$\dot{X}=V_{x}\cos(\psi )-V_{y}\sin(\psi ),$$

(11) $$\dot{Y}=V_{x}\sin(\psi )+V_{y}\cos(\psi ),$$

(12) $$\dot{\psi }=r,$$

(13) $${\dot{V}}_{x}=V_{y}r+\frac{1}{m}\left(F_{xr}-F_{yf}\sin\delta \right),$$

(14) $${\dot{V}}_{y}=-V_{x}r+\frac{1}{m}\left(F_{yf}\cos\delta +F_{yr}\right),$$

(15) $$\dot{r}=\frac{1}{I_{z}}\left(l_{f}F_{yf}\cos\delta -l_{r}F_{yr}\right).$$

### 3.2. V-SLAM Algorithm

Herein, two novel algorithms are introduced into V-SLAM: ISVD, an anti-blurring algorithm, and SFFSD, an algorithm to remove feature outliers [31]. ISVD ensures that the system chooses the less-blurred images. SFFSD removes false matches from the initial putative feature correspondences. In this paper, these two algorithms are employed to enhance the performance (e.g., robustness, efficiency and accuracy) of the V-SLAM module.

#### 3.2.1. ISVD Algorithm

The ISVD algorithm was designed to mitigate the effect of image blurring. ISVD is based on the principal component analysis algorithm, which selects the top k eigenvectors with the highest eigenvalues to represent an image matrix. The quality of an image can be judged by the image singular value.

For an image matrix $I\in R^{m\times n}$, there exists orthogonal matrixes $U\in R^{m\times m}$ and $V\in R^{n\times n}$ that compose $I=UWV^{T}$, where $W=diag(\delta_{1},\delta_{2},\ldots ,\delta_{n})$. $\delta_{1}\geq \delta_{2}\geq \ldots \geq \delta_{n}$ are the non-zero singular values. After the decomposition of the image matrix, the singular value histogram can be established with the non-zero singular values. The definition of $d_{ISVD}$ is given by Equation (16):

(16) $$d_{ISVD}=\frac{\sum_{i}^{n}S_{i}}{n},\;S_{i}=\left\{\begin{array}{l} 1,\begin{array}{ll} & if\;\delta_{i}\geq C_{thres} \end{array}\\ 0,\begin{array}{ll} & if\;\delta_{i}<C_{thres} \end{array} \end{array}\right.,$$

where $\delta_{i}$ is the singular value of W, $C_{thres}$ is a singular value threshold, which was set to 100 in this study.

**Figure 4:**
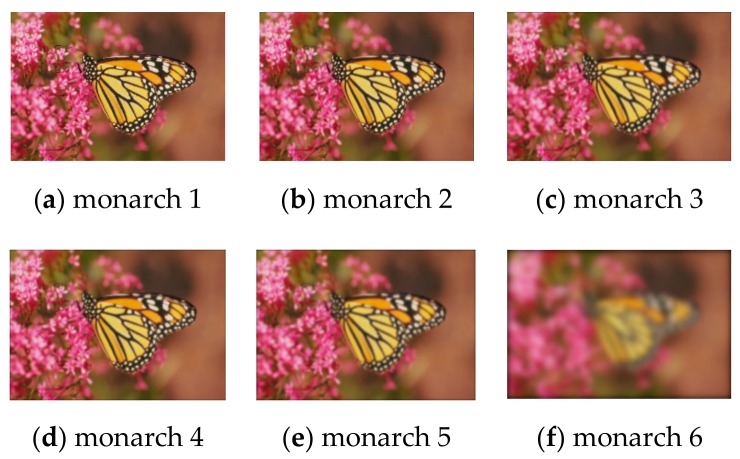
Images with various degrees of blurring from the LIVE2 database. Reproduced from *Zhao, X.; Min, H.; Xu, Z.; Wang, W. An ISVD and SFFSD-based vehicle ego-positioning method and its application on indoor parking guidance. Transportation Research Part C: Emerging Technologies, 2019; 108: 29–48. Copyright © 2019* Elsevier Masson SAS. All rights reserved [33].

**Figure 5:**
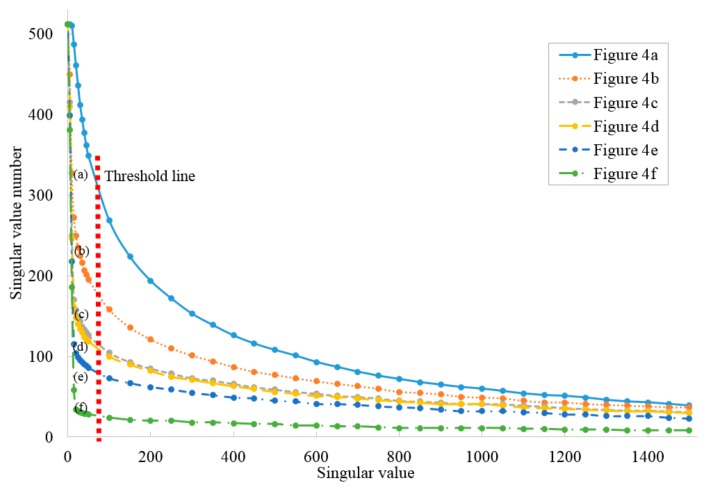
Singular value curves for the images in Figure 4. Reproduced from *Zhao, X.; Min, H.; Xu, Z.; Wang, W. An ISVD and SFFSD-based vehicle ego-positioning method and its application on indoor parking guidance. Transportation Research Part C: Emerging Technologies, 2019; 108: 29–48. Copyright © 2019* Elsevier Masson SAS. All rights reserved [33].

To verify the idea proposed above, Figure 4a–e with increasing degrees of blurring were obtained from the LIVE2 database [32] and are shown in Figure 4. According to ISVD, the image singular value curves of images a–e are depicted in Figure 5, in which the threshold value $C_{thres}$ is marked with a red line.

The calculation of the degree of image blurring using ISVD is summarised in Algorithm 1. The original image matrix is too large to decompose; to speed up this procedure, the anti-blurring process was applied only to key frames, which is more efficient that the corresponding frame-by-frame process.

**Algorithm 1**: Calculation of degree of blurring using image singular value decomposition (ISVD).**Input:** RGB image I **Output:** Blurred degree $d_{ISVD}$ of image I **Initialisation:**
$d_{ISVD}=0$, num=0 1: Convert colour I to grayscale $I_{gray}$ 2: Calculate a singular value $W=diag(\delta_{1},\delta_{2},\ldots ,\delta_{n})$ with singular value decomposition on $I_{gray}$ 3: For $\delta_{i}$ in W 4:  If $\delta_{i}\geq C_{thres}$ 5:   num++ 6:  End 7: End 8: Return blurred degree $d_{ISVD}=num/n$

#### 3.2.2. SFFSD Algorithm

**Figure 6:**
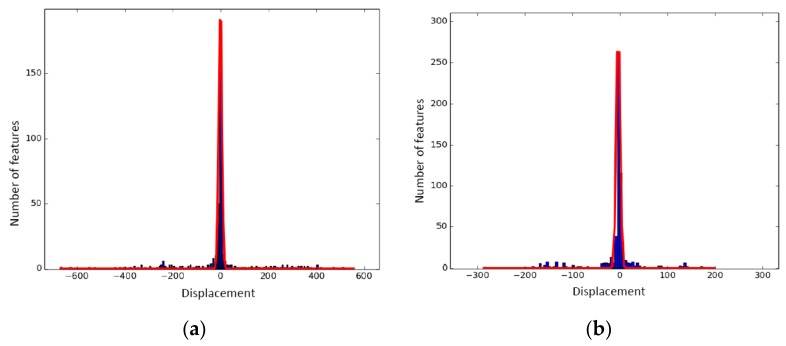
Feature displacement between frames, including inliers and outliers: (**a**) *x*-coordinate and (**b**) *y*-coordinate. Blue: measured displacement error [31]. Red: fitted Laplacian probability distribution function (pdf) with a long tail.

**Figure 7:**
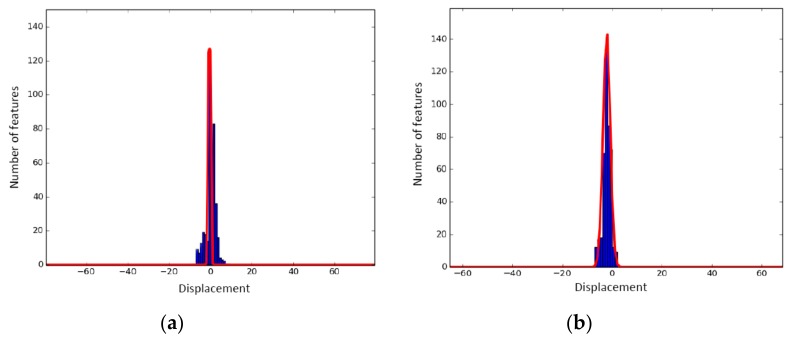
Feature displacement for inliers between frames: (**a**) *x*-coordinate and (**b**) *y*-coordinate. Blue: measured displacement error [32]. Red: fitted Laplacian pdf with a short tail.

In Badino et al. [34], the tracking error in local invariant features between adjacent images was found to follow a Laplacian distribution. Similarly, we found that the feature-matching error of successive image frames $\left(I_{l,i-1},I_{l,i}\right)$ or $\left(I_{r,i-1},I_{r,i}\right)$ along with the feature-tracking error of the left and right matched image frames $\left(I_{l,i-1},I_{r,i-1}\right)$ or $\left(I_{l,i},I_{r,i}\right)$ in the stereo model, conform to a Laplacian distribution. Verified with the experiments, the pixel shift of matched features, including inliers and outliers, along the *x*-axis or *y*-axis was fitted with a Laplacian distribution, which had a long tail, as shown in Figure 6. In contrast, the pixel shift of matched features considering only inliers followed a Laplacian pattern with a short tail. In Figure 7, the line fitting was conducted only with inliers; the fitted line had a short tail and a peak bin. In these two figures, the *x*-axis indicates the displacement in pixels, and the *y*-axis is the number of features collected by histogram statistics.

Based on the above findings, we propose a SFFSD to remove the outliers and retain the inliers. Algorithm 2 briefly describes the SFFSD. Here, we present only the statistic filtering between two candidates matched images *I*_1_ and *I*_2_. In our experiments, the bin number *n* was set to 5. In this case, the corresponding features included 80% inliers. However, the value of *n* was allowed to vary; it depended on the diversity of the surrounding environment. To further refine the matches after statistic filtering, circle matching was employed to verify the matches [35].

**Algorithm 2:** Statistic filtering of feature displacement**Input:** Candidate matched images $I_{1}$, $I_{2}$. **Output:** Good feature matches $VM_{good}$ 1: Detect features $I_{1}$, $I_{2}$ to obtain descriptors $M_{des1}$, $M_{des2}$ and key points $M_{keys1}$, $M_{keys2}$ 2: Match $M_{des1}$, $M_{des2}$ to obtain the original matches $VM_{matches}$ with brute force matcher and hamming distance 3: Calculate the key-points displacements for $VM_{matches}$ in x and y components $\Delta u=u_{1}-u_{2}$, $\Delta v=v_{1}-v_{2}$ 4: Create a 2D histogram with Δu and Δv to confirm the highest bins for mode approximation 5: Use the sample within the radius to perform parameter estimation of the Laplacian distribution in x and y 6: Determine the min and max boundary values to include a certain percentage ratio = 0.9 of inliers, assuming a Laplacian distribution 7: Find the matches $VM_{u\_mats}$ according to the boundary in Step 6 8: Repeat Steps 6 and 7 to find the matches $VM_{v\_mats}$ 9: Calculate the common element $V_{com}$ from $VM_{u\_matches}$ and $VM_{v\_matches}$ 10: For id in $VM_{matches}$ 11:  If id in $V_{com}$ 12:   Pushback the corresponding element into $VM_{good}$ 13:  End 14: End

### 3.3. V-SLAM Track and GPS Track Calibration

In this part, the V-SLAM track $p_{i}$ is used to the correct GPS trajectory $q_{i}$ via regression analysis. This process included three main steps: (1) coordinate system conversion, (2) time alignment and improved iterative closest point (ICP) calibration, and (3) inverse coordinate system conversion.

In common applications, the GPS value is defined in the geocentric coordinate system. To perform regression analysis, the GPS value needs to be converted to the local spatial coordinate system. In this paper, we converted from the geocentric coordinate system to the “east, north, up” coordinate system via the universal transverse mercator (UTM) projection, as shown in Equations (17) and (18):

(17) $$x_{local}=k_{0}\left(M+N\tan y\left(\frac{x^{2}}{2}+(5-T+9C+4C^{2})\right)\frac{x^{4}}{24}\right.\left.+(61-58T+T^{2}+600C-330e_{1}^{2})\frac{x^{6}}{720}\right),$$

(18) $$y_{local}=k_{0}N\left(x+\left((1-T+C)\times \frac{x^{3}}{6}\right)\right.\left.+(5-18T+T^{2}+72C-58e_{1}^{2})\times \frac{x^{5}}{120}\right),$$

where:

(19) $$r=\frac{y\cdot \pi }{180},$$

(20) $$T=\tan^{2}r,$$

(21) $$e_{1}=\frac{\sqrt{a^{2}-b^{2}}}{a},$$

(22) $$e_{2}=\frac{\sqrt{a^{2}-b^{2}}}{b},$$

(23) $$C=e_{2}^{2}\cos^{2}r,$$

(24) $$A=\frac{(x-L_{0})\cdot \pi \cos r}{180},$$

(25) $$N=\frac{a}{\sqrt{1-e_{1}^{2}\sin^{2}r}},$$

(26) $$m_{0}=1-\frac{e_{1}^{2}}{4}-\frac{3e_{1}^{4}}{64}-\frac{5e_{1}^{6}}{256},$$

(27) $$m_{1}=-\frac{3e_{1}^{2}}{8}-\frac{3e_{1}^{4}}{32}-\frac{45e_{1}^{6}}{1024},$$

(28) $$m_{2}=\frac{15e_{1}^{4}}{256}+\frac{45e_{1}^{6}}{1024},$$

(29) $$m_{3}=-\frac{35e_{1}^{6}}{3072},$$

(30) $$M=a(m_{0}r+m_{1}\sin(2r)+m_{2}\sin(4r)+m_{3}\sin(6r)),$$

where *k_0_* is a scale factor (0.9996 in this paper); *x* and *y* are the longitude and latitude, respectively; *a* (6,378,245.0) and *b* (6,356,863.0188) are the equatorial radius and polar radius, respectively; *L_0_* is the central meridian; *e*_1_ is the first eccentricity (0.0818191908); *f_e_* is the false easting value (500,000); and *f_n_* is the false northing value (10,000,000).

We improved the original ICP by accounting for the weight *w_i_* at each timestamp. First, we calculated the centroids of three-dimensional points generated using the V-SLAM algorithm and the centroids of GPS after the coordinate conversion. Next, the covariance matrix between the measurements of V-SLAM and GPS was calculated. A singular value decomposition was carried out to obtain the rotation and translation matrices. This process is summarised in Algorithm 3.

**Algorithm 3:** Improved weighted iterative closest point (ICP)**Input:** V-SLAM track $p_{i}$, GPS track $q_{i}$, weights on timestamps $\left\{\left.w_{i}\right|w_{i}\geq 0\right\}$, i=1,…,N **Output:** Rotation matrix and translation vector that minimises ${\sum_{i=1}^{N}w_{i}\left\|q_{i}-\left(Rp_{i}+t\right)\right\|}^{2}$ 1: Centroids $\bar{p}=\left(\sum_{i=1}^{N}w_{i}p_{i}\right)/\left(\sum_{i=1}^{N}w_{i}\right)$, $\bar{q}=\left(\sum_{i=1}^{N}w_{i}q_{i}\right)/\left(\sum_{i=1}^{N}w_{i}\right)$ 2: Centred vectors $x_{i}=q_{i}-\bar{q}$, $y_{i}=p_{i}-\bar{p}$ 3: Covariance matrix $S=XWY^{T}$, where X and Y have $x_{i}$ and $y_{i}$ as columns, respectively, and W is a diagonal matrix with $w_{i}$ on the diagonal 4: Singular value decomposition $S=U\Sigma V^{T}$ 5: $R=UV^{T}$ and $t=\bar{q}-R\bar{p}$ 6: V-SLAM track after calibration in global coordinates ${p^{\prime }}_{i}=Rp_{i}+t$

The traditional ICP method based on least squares is not robust against outliers (i.e. this method tends to be affected by bad GPS signals, which are common in challenging outdoor environments). To improve the robustness of calibration, least absolute deviations is used to eliminate the effect of outliers. The problem can be solved via iteratively re-weighted least squares. The result of iteration converges to least absolute deviations; that is, $\sum_{i=1}^{N}s_{i}\left\|q_{i}-\left(Rp_{i}+t\right)\right\|$ is minimised, where $s_{i}=\left\|p_{i}-p_{i-1}\right\|$. The final weights are used as a measurement of credibility, which is part of the weight assigned to the timestamps. The credibility for timestamp $c_{i}$ is calculated using:

(31) $$c_{i}=\frac{1}{\max\left(\phi ,\left\|q_{i}-\left(Rp_{i}+t\right)\right\|\right)},$$

where *R* and *t* are the rotation matrix and translation vector obtained in Algorithm 3, respectively; and *ϕ* is the credibility distance bound. We updated the weight using $w_{i}=s_{i}c_{i}$. The loop continues until it meets the condition given by ${\sum_{i=1}^{N}w_{i}\left\|q_{i}-\left(Rp_{i}+t\right)\right\|}^{2}<e$.

The last operation in this process is the inverse coordinate system conversion (i.e., inverse UTM projection). At this point, we could obtain the accurate global position information as follows:

(32) $$x=L_{0}+\frac{1}{\cos B_{f}}\left[D-(1-2T_{f}+C_{f})\frac{D^{3}}{6}\right.\left.+(5+28T_{f}+24T^{2}{}_{f}-2C_{f}-3C^{2}{}_{f}+8{e^{\prime \prime }}^{2})\frac{D^{2}}{120}\right],$$

(33) $$y=B_{f}-\frac{N_{f}\tan B_{f}}{R_{f}}\left[\frac{D^{2}}{2}-(5+3T_{f}+10C_{f}-4C^{2}{}_{f}-9{e^{\prime \prime }}^{2})\frac{D^{4}}{24}\right.\left.+(61+90T_{f}+45T^{2}{}_{f}+298C_{f}-3C^{2}{}_{f}-252{e^{\prime \prime }}^{2})\frac{D^{6}}{720}\right],$$

where:

(34) $$e=\frac{1-b/a}{1+b/a},$$

(35) $$M_{f}=\frac{x_{local}}{k_{0}},$$

(36) $$D=\frac{y_{local}}{k_{0}N_{f}},$$

(37) $$T_{f}=\tan^{2}(B_{f}),$$

(38) $$C_{f}=e_{2}^{2}\cos^{2}(B_{f}),$$

(39) $$N_{f}=\frac{a}{\sqrt{1-e_{1}^{2}\sin^{2}(B_{f})}},$$

(40) $$R_{f}=\frac{a(1-e_{1}^{2})}{\sqrt{{\left(1-e_{1}^{2}\sin^{2}(B_{f})\right)}^{3/2}}},$$

(41) $$\phi =\frac{M_{f}}{a(1-e_{1}^{2}/4-3e_{1}^{4}/64-5e_{1}^{6}/256)},$$

(42) $$B_{f}=\phi +\left(\frac{3e}{2}-\frac{27e^{3}}{32}\right)\sin(2\phi )+\left(\frac{21e^{2}}{16}-\frac{55e^{4}}{32}\right)\sin(4\phi )+\frac{151e^{3}}{96}\sin(6\phi ).$$

### 3.4. Interacting Multiple Model (IMM) Filter

The IMM filter is designed to calculate the weight of each model under changing external driving conditions and estimate the vehicle position. The IMM filter is composed of four parts: (1) interaction, (2) two extended Kalman filters (one for the kinematic vehicle model and a second for the dynamic vehicle model), (3) a model probability update and (4) the fusion of state and covariance estimation from the two models.

#### 3.4.1. Interaction

The states from the kinematic and dynamic vehicle models are mixed with each other using a predicated probability. The mixing probability $u_{k-1}^{j|i}$ is expressed as:

(43) $$u_{k-1}^{j|i}=\pi_{ji}u_{k-1}^{j}/u_{k|k-1}^{i}\;\left(i,j=1,2\right),$$

where $u_{k-1}^{j}$ is the probability of the model *j*; and $\pi_{ji}$ is the probability for the transition from model *j* to model *i*, which is calculated based on a prior knowledge using a statistical method [32]. The index *i*, *j* = 1 refers to the kinematic vehicle model, while *i*, *j* = 2 represents the dynamic vehicle model. The predicated model probability $u_{k|k-1}^{i}$ is given as:

(44) $$u_{k|k-1}^{i}=\sum_{j=1}^{2}\pi_{ji}u_{k-1}^{j}\;\left(i=1,2\right).$$

The mixing state ${\bar{X}}_{k-1|k-1}^{i}$ and their covariance ${\bar{P}}_{k-1|k-1}^{i}$ of the model *i* are respectively computed using:

(45) $${\bar{X}}_{k-1|k-1}^{i}=\sum_{j=1}^{2}u_{k-1}^{j|i}{\hat{X}}_{k-1|k-1}^{j}\;\left(i=1,2\right),$$

(46) $${\bar{P}}_{k-1|k-1}^{i}=\sum_{j=1}^{2}u_{k-1}^{j|i}\left[{\hat{P}}_{k-1|k-1}^{j}+\Delta {\bar{X}}_{k-1}^{ij}{\left(\Delta {\bar{X}}_{k-1}^{ij}\right)}^{T}\right]\;\left(i=1,2\right).$$

In Equations (45) and (46), $\Delta {\bar{X}}_{k-1}^{ij}={\bar{X}}_{k-1|k-1}^{i}-{\hat{X}}_{k-1|k-1}^{j}$, where ${\hat{X}}_{k-1|k-1}^{j}$ and ${\hat{P}}_{k-1|k-1}^{j}$ are the state and covariance of the model j in the previous step, respectively.

#### 3.4.2. Extended Kalman Filter

**Figure 8:**
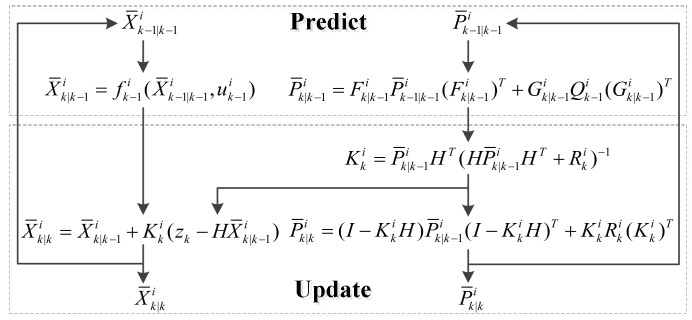
Flowchart of the extended Kalman filter in the interacting multiple model (IMM).

The extended Kalman filter was adopted to predict and update the state and covariance of each model. The prediction and update steps were carried out based on the equations shown in Figure 8. The state vector is represented as $X_{k}=(x_{k},y_{k},h_{k},\psi_{k},\beta_{k},v_{k})$, while the input vector is $u_{k}=(v_{k\_wheel},\delta_{k})$.

In Figure 8, $F_{k|k-1}^{i}={\left.\frac{\delta f_{i}}{\delta x}\right|}_{x={\bar{X}}_{k-1|k-1}^{i}}(i=1,2)$ and $G_{k-1}^{i}$ are the Jacobian matrices of the process function $f_{k-1}^{i}(\cdot )$ with respect to ${\bar{X}}_{k-1|k-1}^{i}$ and $u_{k-1}^{i}$, respectively; H is the Jacobian matrix of the measurement function; $Q_{k-1}^{i}$ is the covariance matrix of the process noise; $K_{k}^{i}$ is the Kalman filter gain associated with the measurement sensors; and $R_{k}^{i}$ is the covariance matrix of the measurement noise.

In the prediction step, the state ${\bar{X}}_{k|k-1}^{i}$ and covariance ${\bar{P}}_{k|k-1}^{i}$ are calculated with the mixing state ${\bar{X}}_{k-1|k-1}^{i}$ and their covariance ${\bar{P}}_{k-1|k-1}^{i}$. In the update step, the corrected state ${\bar{X}}_{k|k}^{i}$ and covariance ${\bar{P}}_{k|k}^{i}$ are updated with the calibration result with the GPS and V-SLAM module.

#### 3.4.3. Model Probability Update

Each model probability is updated based on the model innovation. Assuming Gaussian statistics, the probability of model *i* at time *k* for the observation is calculated using:

(47) $$\Lambda_{k}^{i}=\frac{\exp\left\{-0.5\left[{\left(z_{k}-H{\bar{X}}_{k|k-1}^{i}\right)}^{T}{\left(S_{k}^{i}\right)}^{-1}(z_{k}-H{\bar{X}}_{k|k-1}^{i})\right]\right\}}{\sqrt{\left|2\pi S_{k}^{i}\right|}}\;(i=1,2),$$

with

(48) $$u_{k}^{i}=\frac{\Lambda_{k}^{i}u_{k|k-1}^{i}}{\sum_{j=1}^{2}\Lambda_{k}^{j}u_{k|k-1}^{j}}\;\left(i=1,2\right),$$

where $S_{k}^{i}$ is the residual covariance matrix.

#### 3.4.4. Estimation Fusion

The output state ${\bar{X}}_{k|k}$ and its covariance ${\bar{P}}_{k|k}$ are computed according to the Gaussian mixture equation:

(49) $${\bar{X}}_{k|k}=\sum_{i=1}^{2}u_{k}^{i}{\bar{X}}_{k|k}^{i},$$

with

(50) $${\bar{P}}_{k|k}=\sum_{i=1}^{2}u_{k}^{i}\left[{\bar{P}}_{k|k}^{i}+\Delta {\bar{X}}_{k}^{i}{\left(\Delta {\bar{X}}_{k}^{i}\right)}^{T}\right],$$

where $\Delta {\bar{X}}_{k}^{i}={\bar{X}}_{k|k}-{\bar{X}}_{k|k}^{i}$.

## 4. Experiment and Results

**Figure 9:**
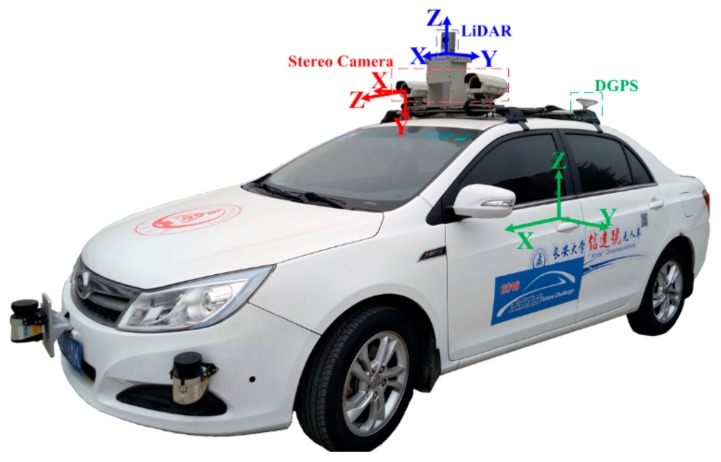
Autonomous vehicle platform. Reproduced from *Zhao, X.; Min, H.; Xu, Z.; Wang, W. An ISVD and SFFSD-based vehicle ego-positioning method and its application on indoor parking guidance. Transportation Research Part C: Emerging Technologies, 2019; 108: 29-48. Copyright © 2019* Elsevier Masson SAS. All rights reserved [33].

Figure 9 shows the experimental platform. An autonomous vehicle, “Sinda,” was equipped with a high-resolution stereo camera rig (Basler Ace1600 GigE, Basler, Ahrensburg, Germany, image size 960 pixels × 720 pixels), LiDAR (Velodyne HDL-32E, Velodyne, CA, USA) and GPS (NovAtel OEM718D, NovAtel, Calgary, Canada) and a micro-electro-mechanical system (MEMS)-based IMU (ADIS16465, Analog Devices, Norwood). The parameters of the vehicle are shown in Table 1.

### 4.1. Simulation

**Figure 10:**
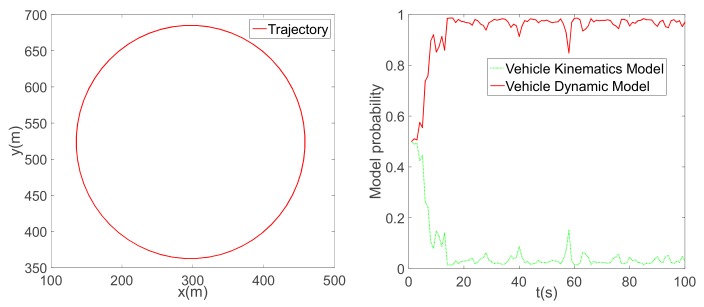
A circular trajectory and vehicle model probability.

**Figure 11:**
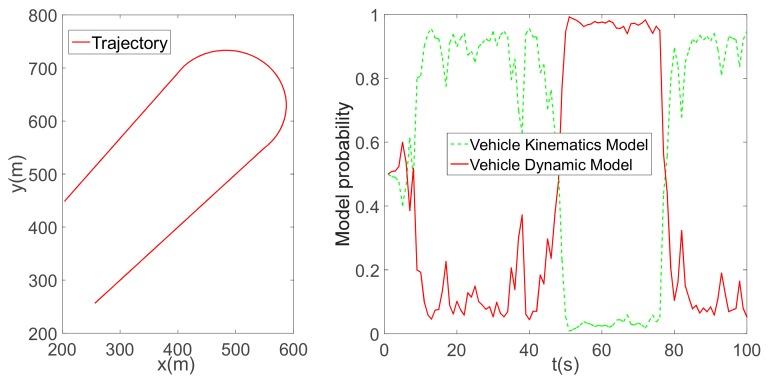
A U-turn trajectory and vehicle model probability.

To ensure consistency with the simulation, we applied the same vehicle parameters in the experiment. To verify the reasonableness of the established vehicle model, two typical scenes were simulated: a circular trajectory (Figure 10) and a U-turn trajectory (Figure 11). In the first simulation, the vehicle speed was set to approximately 55 km/h. Due to a high level of tyre slip, the dynamic vehicle model was adopted for position estimation. In the second simulation, the vehicle speed was maintained at approximately 45 km/h. In a straight line, the vehicle kinematic model was consistent with the assumption of no tyre slip; however, this assumption broke down for sharp turns. When the vehicle drove in a straight line, the model probability returned from the dynamic vehicle model to the kinematic model. The simulations demonstrate that the IMM could adapt to several driving conditions, which is vital for application in real-world situations.

### 4.2. Benchmark Dataset

**Figure 12:**
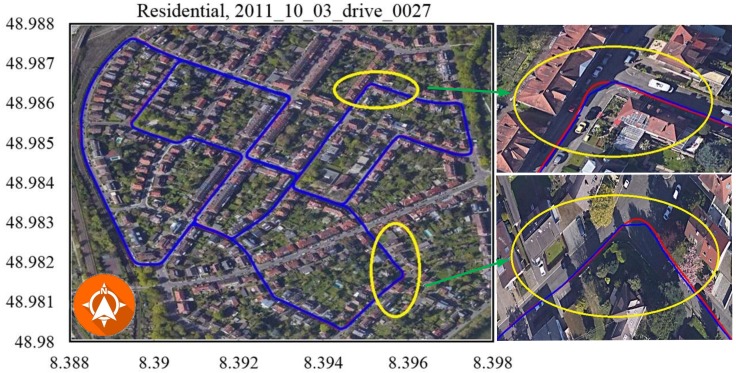
Residential, 2011_10_03_drive_0027. There was an error in the trajectory because the car moved into a cluttered environment, and the GPS signal was blocked or had a multipath effect.

**Figure 13:**
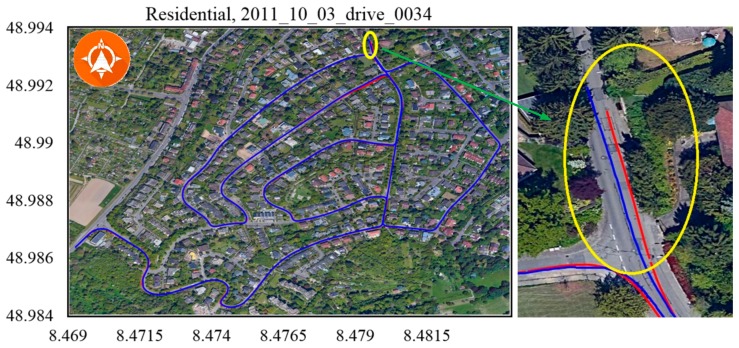
Residential, 2011_10_03_drive_0034. The ending point of the blue line tended to drift off lane, while the red one kept inside the lane.

Due to a lack of in-vehicle sensor data in the KITTI benchmark dataset, verification experiments were conducted using only camera (Point Grey Flea 2 FL2-14S3C-C, FLIR System, Arlington, VA, USA) and GPS (OXTS RT3003, OXTS, Middleton Stoney, UK) data (No.2011_10_03_drive_0027, Figure 12; No.2011_10_03_drive_0034, Figure 13) from residential scenarios. When the vehicle made a sharp turn or moved through a cluttered environment, the GPS-based trajectory (marked by the blue line) had an unacceptable positioning error, while the trajectory of our method (marked in red) was closer to the real trajectory.

**Figure 14:**
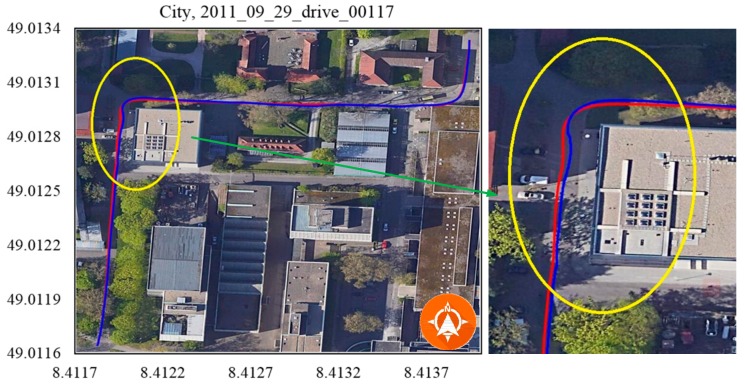
City, 2011_09_29_drive_00117. There was a big error in the marked area because of the tall buildings on two sides of the road.

**Figure 15:**
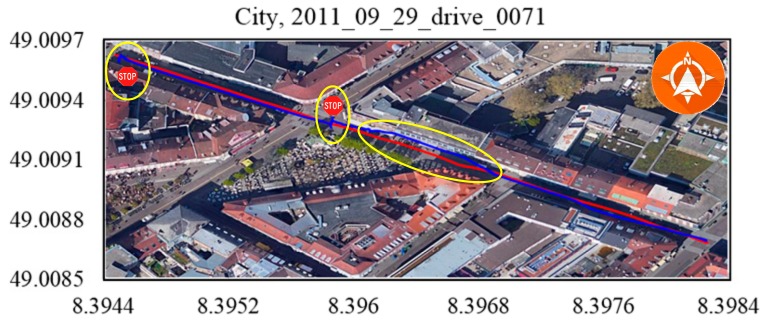
City, 2011_09_29_drive_0071. The GPS signal was blocked when the vehicle passed through city roads with tall buildings.

**Figure 16:**
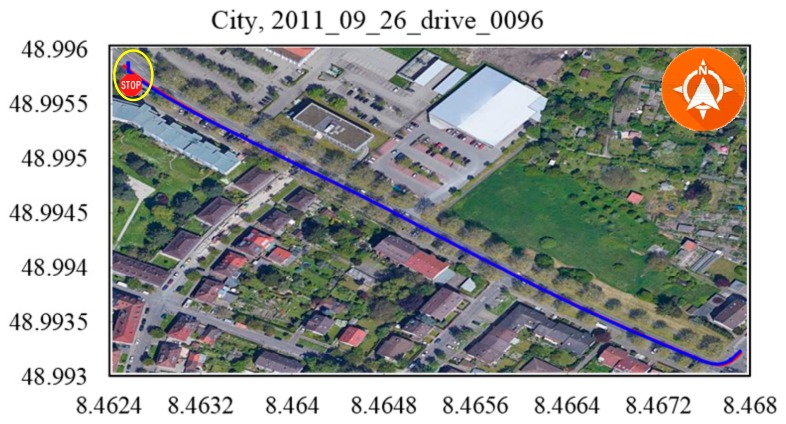
City, 2011_09_26_drive_0096. At the position of the stop sign, the GPS signal was not very stable.

To our knowledge, the GPS signal will be blocked or reflected by tall buildings or thick trees around the road. The data 2011_09_29_drive_00117 (Figure 14), 2011_09_29_drive_0071 (Figure 15) and 2011_09_26_drive_0096 (Figure 16) of city scenarios verified the accuracy and stability of the positioning methods well. The instability was magnified when the vehicle stopped to wait at the traffic lights. There was a sudden jump in the GPS coordinate while the proposed method was stable and accurate. We denoted the vehicle-stop position with a stop sign on the trajectory. The abscissa axis and ordinate axis denotes the longitude and latitude values, respectively.

After comparisons, the basic information is shown in Table 2. The distance of the ground truth from OXTS RT3003 on the KITTI benchmark was different from the distance estimated using our proposed method. The extra distance was naturally caused by losing the GPS signal or a multipath effect. To specify the relative errors between these methods, the root mean square errors (RMSEs) are shown in below Table 2.

### 4.3. Real Data

To evaluate the accuracy and reliability of the developed algorithm, outdoor tests were conducted using various methods: normal DGPS (green), DGPS/IMU (cyan), DGPS/IMU/in-vehicle sensors (blue) and DGPS/IMU/in-vehicle sensors/SLAM calibration (red). Moreover, DGPS/IMU/in-vehicle sensors was regarded as the “ground truth” to calculate the RMSE of different methods. The experiments conducted with real data were divided into three parts. First, experiments were conducted in bad GPS conditions corresponding to the most challenge case regularly encountered by an autonomous vehicle. Second, a short-distance experiment including different driving behaviours was conducted to test the adaptability of the system. These driving behaviours were selected to introduce unstable factors into the vehicle positioning system. Finally, a long-distance trajectory experiment was performed as a full test. The proposed global optimisation model eliminated the cumulative error derived from the incremental characteristic of the methods (e.g., odometry and DR methods). The abscissa axis and ordinate axis denote the longitude and latitude values, respectively.

#### 4.3.1. Bad GPS Conditions

**Figure 17:**
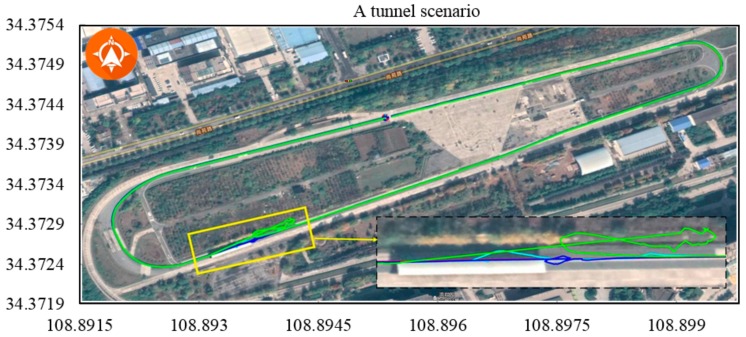
Tunnel scenario. To highlight the stability of the proposed positioning method, the vehicle moved slowly and stopped briefly at the end of the tunnel. The GPS signal was blocked or reflected. All GPS-based methods were affected, resulting in drift of several meters. In contrast, the proposed method was stable and accurate.

**Figure 18:**
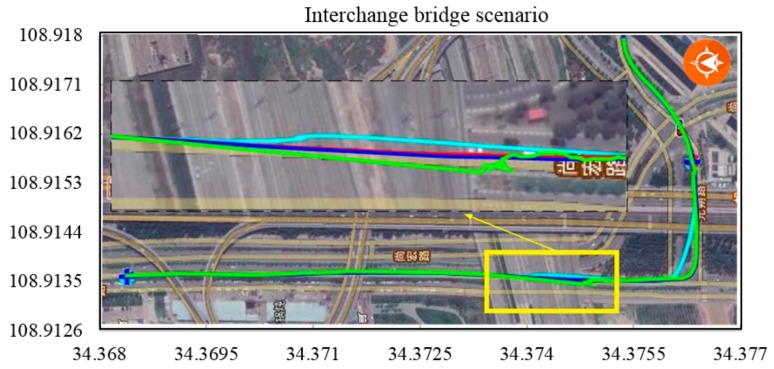
Interchange bridge scenario. The GPS signal was clearly blocked when the vehicle passed through the interchange bridge. Because of the IMU and in-vehicle sensors, the DGPS/IMU and DGPS/IMU/in-vehicle sensors methods could handle this challenge. In contrast, the DGPS-only method was inaccurate, as marked with a rectangle (The abscissa axis and ordinate axis denote the latitude and longitude values, respectively).

The most challenging driving conditions for GPS localisation are those where the GPS signal is blocked or reflected. We experimentally tested the special case where an autonomous vehicle stopped in a place where the GPS signal was blocked or reflected by buildings, tunnels or bridges, as shown in Figure 17 and Figure 18. In these cases, the GPS signal was not stable or precise, and the radius of the circle that covered all GPS points was large when using normal DGPS, DGPS/IMU and DGPS/IMU/in-vehicle sensors. Not surprisingly, the proposed method exhibited absolute stability resulting from the local accuracy of the SLAM module. The comparison results in the bad GPS condition are shown in Table 3.

#### 4.3.2. Short-Distance Trajectory with Different Driving Behaviours

**Figure 19:**
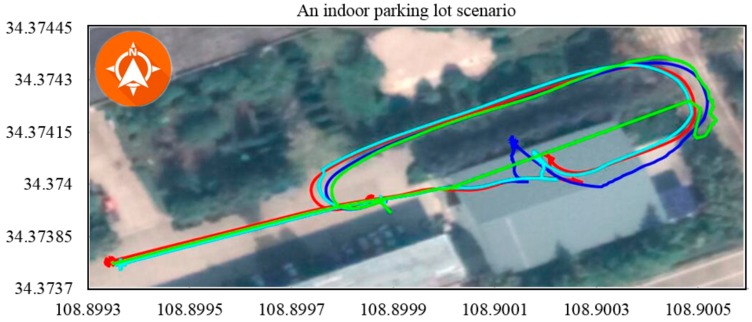
Scenario where the vehicle passes through an indoor parking lot and reverses into an empty parking space. The DGPS-only method lost the signal. The DGPS/IMU and DGPS/IMU/in-vehicle sensors methods resulted in a drift of approximately 6 m, which is unacceptable for autonomous vehicles.

**Figure 20:**
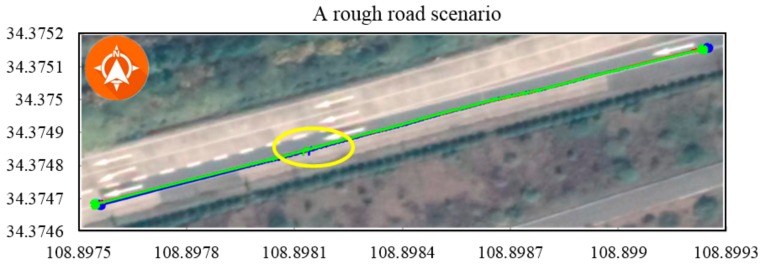
Scenario involving violent shaking on a rough road. The shaking movement affected the IMU-based or in-vehicle sensor-based positioning, and the maximum error was approximately 1.2 m, as indicated by the yellow circle.

To exhaustively explore the adaptability of the system, experiments involving different driving behaviours (e.g., quick starts and stops, sharp turns, reversing the car and driving in challenging conditions, such as on a rough road or in an indoor parking lot) were conducted. Quick starts and stops along with sharp turns resulted in tyre slip for vehicles driving on road surfaces with low adhesion coefficients. As shown in Figure 19, the vehicle passed through an indoor parking lot and reversed into an empty parking space to test the algorithm performance in a realistic scenario. With the exception of the proposed method, all positioning methods performed badly. Violent shaking introduced error in the IMU-based method, as shown in Figure 20. Unexpectedly, the DGPS-only method performed almost equal with IMU-based positioning methods. The comparison results of the short-distance trajectory with various driving behaviors are shown in Table 4.

#### 4.3.3. Long-Distance Trajectory in a Cluttered Environment

**Figure 21:**
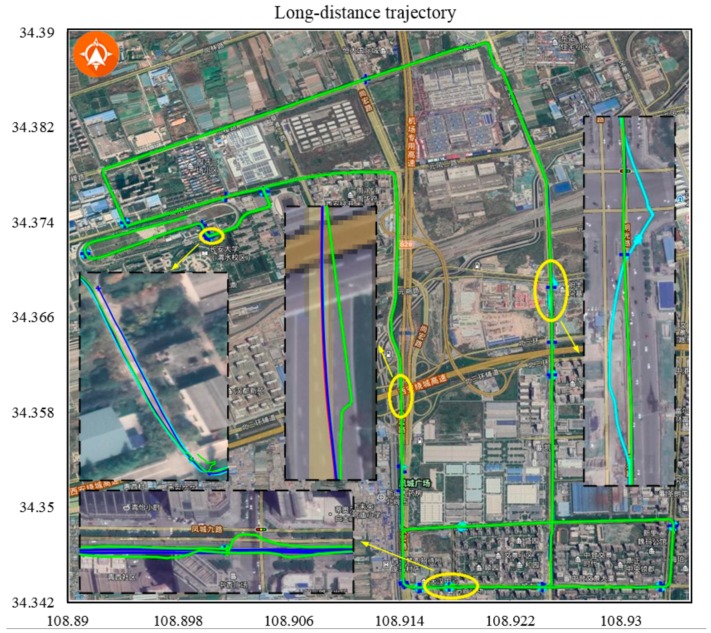
A 21-km trajectory that lasted about 47 min. All the methods had relatively poor performance except the proposed calibration method.

A long-distance experiment including many of the scenarios found on urban and suburban roads (e.g., traffic lights and waiting zones) was performed as a final complete test. The main purpose of the long-distance experiment was to evaluate the effect of the SLAM module on the positioning result. Because SLAM is a locally accurate and incremental positioning method, cumulative error was inevitable. The proposed global optimisation strategy could eliminate this error, as shown in Figure 21. After the vehicle stopped at the stop line, we verified that the positioning point and the stop line on the map were coincident. The Comparison results of long-distance trajectory in a challenging environment are shown in Table 5.

## 5. Conclusions

The positioning and navigation system is a critical component of an autonomous vehicle. We have proposed a real-time, precise and low-cost vehicular positioning method. Common GPS and MEMS-based IMUs are affordable for use in commercial autonomous vehicles. For vehicles equipped with automatic braking systems and electronic stability control, the steering angle and the speeds of the four wheels can be directly obtained via the vehicle’s CAN bus. Thus, the proposed strategy can be applied in autonomous vehicles.

The main contribution of this paper is the combination of V-SLAM for local coordinates and GPS for global coordinates considering their complementary properties. ISVD and SFFSD algorithms were proposed to ensure an accurate and reliable V-SLAM. The fusion was performed using an improved weighted ICP and least absolute deviations. The appropriate vehicle or kinematic model was selected according to the current state of the moving vehicle.

The performance of the proposed method was sufficiently verified by simulations, benchmark tests and experimental scenarios. The relationship between the driving state and vehicle model was analysed through various simulations. Using the KITTI benchmark database, we compared the ground-truth data with our proposed calibration method. The proposed method performed better than conventional methods in some special scenarios. In the experimental scenarios, an autonomous vehicle was driven through challenging environments (e.g., a tunnel and interchange bridge). The results showed that the developed algorithm satisfied the accuracy and reliability requirements for autonomous vehicle positioning and navigation. In future work, the algorithm will be extended to consider more extreme driving conditions, such as icy pavements and long tunnels. The developed positioning system will be applied to the applications of an autonomous vehicle indoor parking system or truck platoon. In addition, to promote the development and application of embedded positioning systems, the efficiency should be comprehensively considered.

## Figures and Tables

**Table 1:** Parameter of the experiment vehicle.

Parameter	Symbol	Value	Unit
Vehicle mass	*m*	1395	kg
Yaw moment of inertial	*I_z_*	4192	kg·m^2^
The distance from *G* to the front wheel *F*	*a*	1.04	m
The distance from *G* to the rear wheel *R*	*b*	1.62	m
The height of center of gravity	*H*	0.54	m
Wheel track	*d*	1.52	m
Rolling resistance coefficient	*f*	0.02	_
The rolling radius of the tyres	*r*	0.335	m
Frontal area	*A*	1.8	m^2^
The coefficient of air resistance	*C_D_*	0.343	_
The density of air	*ρ*	1.206	kg/m^3^

**Table 2:** The positioning results on KITTI benchmark.

Scenarios	No.	Duration (s^−1^)	Distance (m^−1^) OXTS RT3003/Proposed Method	RMSE (m^−1^)
Residential	Figure 12	593.24	3734.375/3727.332	0.645
	Figure 13	584.79	5066.272/5051.748	1.265
	Figure 14	67.08	392.705/391.232	0.959
City	Figure 15	130.90	341.491/333.710	1.868
	Figure 16	48.61	401.604/397.509	0.858

**Table 3:** Comparison results in bad GPS condition.

No.	Duration (s)	Methods	Distance (m)	RMSE (m)
Figure 17	264.796	GPS	1685.755	13.137
		GPS + IMU	1582.860	4.880
		GPS + IMU + CAN	1589.760	-
		Proposed method	1570.616	1.564
Figure 18	158.069	GPS	1066.827	73.160
		GPS+IMU	1045.851	1.765
		GPS+IMU+CAN	1044.588	-
		Proposed method	1038.825	1.229

**Table 4:** Comparison results of the short-distance trajectory with various driving behaviors.

No.	Duration	Methods	Distance	RMSE
Figure 19	76.105	GPS	218.940	7.105
		GPS + IMU	216.019	4.657
		GPS + IMU + CAN	218.946	-
		Proposed method	213.382	2.341
Figure 20	60.885	GPS	136.538	1.491
		GPS + IMU	142.303	1.139
		GPS + IMU + CAN	131.749	-
		Proposed method	130.997	0.941

**Table 5:** Comparison results of long-distance trajectory in a challenging environment.

No.	Duration	Methods	Distance	RMSE
Figure 21	2843.571	GPS	21,371.167	180.517
		GPS + IMU	21,255.434	11.436
		GPS + IMU + CAN	21,237.723	-
		Proposed method	21,212.237	1.165
